# Conclusions about Niche Expansion in Introduced *Impatiens walleriana* Populations Depend on Method of Analysis

**DOI:** 10.1371/journal.pone.0015297

**Published:** 2010-12-29

**Authors:** Lisa Mandle, Dan L. Warren, Matthias H. Hoffmann, A. Townsend Peterson, Johanna Schmitt, Eric J. von Wettberg

**Affiliations:** 1 Ecology and Evolutionary Biology, Brown University, Providence, Rhode Island, United States of America; 2 Department of Botany, University of Hawaii, Manoa, Honolulu, Hawaii, United States of America; 3 Section of Integrative Biology, University of Texas at Austin, Austin, Texas, United States of America; 4 Institut für Geobotanik und Botanischer Garten, Martin-Luther-Universität Halle – Wittenberg, Halle (Saale), Germany; 5 Biodiversity Institute, University of Kansas, Lawrence, Kansas, United States of America; 6 Department of Biological Sciences, Florida International University, Miami, Florida, United States of America; 7 Center for Tropical Plant Conservation, Fairchild Tropical Botanical Garden, Coral Gables, Florida, United States of America; Trinity College Dublin, Ireland

## Abstract

Determining the degree to which climate niches are conserved across plant species' native and introduced ranges is valuable to developing successful strategies to limit the introduction and spread of invasive plants, and also has important ecological and evolutionary implications. Here, we test whether climate niches differ between native and introduced populations of *Impatiens walleriana*, globally one of the most popular horticultural species. We use approaches based on both raw climate data associated with occurrence points and ecological niche models (ENMs) developed with Maxent. We include comparisons of climate niche breadth in both geographic and environmental spaces, taking into account differences in available habitats between the distributional areas. We find significant differences in climate envelopes between native and introduced populations when comparing raw climate variables, with introduced populations appearing to expand into wetter and cooler climates. However, analyses controlling for differences in available habitat in each region do not indicate expansion of climate niches. We therefore cannot reject the hypothesis that observed differences in climate envelopes reflect only the limited environments available within the species' native range in East Africa. Our results suggest that models built from only native range occurrence data will not provide an accurate prediction of the potential for invasiveness if applied to areas containing a greater range of environmental combinations, and that tests of niche expansion may overestimate shifts in climate niches if they do not control carefully for environmental differences between distributional areas.

## Introduction

The capacity to predict where a species is likely to become invasive could provide valuable insight into population and community ecology, as well as inform efforts towards remediation of the effects of introduced species. With increasingly fine-scale datasets and improved computational capabilities, visualizing and analyzing these possibilities is increasingly feasible [1]–[3]. Although plant distributions are influenced by a combination of abiotic factors, biotic interactions, and dispersal abilities [4], climate is considered a critical determinant of species' ranges, at least on broad spatial scales [5]–[7]. The degree of climate match between native and introduced ranges has been shown to be significant in determining potential distributions of introduced plants [8]–[10]. This climate-matching approach to understanding and predicting potential geographic ranges of invasive species is generally addressed via ecological niche models (ENMs) built by integrating occurrence data with climate data [2].

Many factors might restrict a species from occupying a particular area. The fundamental niche [4], [11] represents the complete set of environmental conditions (e.g. climate, soil type) under which a species can persist. The realized niche – the environmental conditions under which a species is found to occur – is the subset of the species' fundamental niche from which it is not excluded by biotic interactions. Both of these niches can be modified further by the potentially more limited suite of environments *actually represented* on the landscape of the distributional areas, leading us to recognize two further concepts: the “existing fundamental niche” and the “existing realized niche,” as the portions of the two niches that are actually manifested on relevant landscapes [12]. Species may additionally fail to occupy locations with suitable conditions owing to limited dispersal ability.

The degree to which climate niches vary or are conserved across species' native and introduced ranges is a question of considerable current interest in invasion biology [13]–[17]. In addition to ecological and evolutionary implications, this question is important to developing proactive policies to limit the introduction and spread of invasive plant species. If plant species' climate niches are conserved across native and introduced ranges, then climate matching between native and introduced ranges can provide estimates of where introduced species are likely to spread and persist. A lack of climate match would then provide a good indication that the species is unlikely to become invasive, and resources aimed at preventing introduction could be directed elsewhere.

Although studies normally focus on invaded regions successfully predicted from native ranges [e.g. 2], [18], [19]–[21], ENMs calibrated on a species' native range may fail to predict the introduced range for multiple reasons. Models that are under-parameterized or over-parameterized may provide erroneous predictions. False positives may be produced when environmental factors not considered in the analysis preclude establishment and spread of the introduced species in an otherwise suitable climate. On the other hand, false negatives may result from ENMs that have been parameterized using too many environmental variables [16], [22]. In these cases, the breadth of the modeled ecological niche may be narrowed artificially by inclusion of factors that do not in actuality limit the species' establishment [23].

Ecological niche models may also fail to predict a species' introduced range as a result of genuine differences between the climate envelopes of native and introduced populations [13]–[15]. Such differences would be of great interest, but have proven difficult to demonstrate robustly [23]. When climate niches occupied by native and introduced populations do differ, several possible explanations are available: (1) populations may fail to occupy their entire fundamental niche owing to different biotic interactions or dispersal limitations, (2) niches may differ owing to distinct climate regimes on the particular landscapes [15], [18], or (3) the species' ecological niche may truly differ between native and introduced populations, such that introduced populations can persist in a different range of environmental conditions than populations from the native range [14]. These differences might result from interbreeding between individuals of different geographic origins within the introduced range, local adaptation, or genetic drift [15], [24]. In instances where the niche of introduced populations cannot be estimated from native populations, a species' invasive potential may be drastically underestimated by ENMs calibrated only with native range data [13], [14], [18]. The possibility of disregarding a potential invader erroneously on the basis of climate mismatches is thus a serious concern.

Climate matching as a necessary precursor to invasion has been incorporated into policies for importing plant material. For example, climate match comprises a substantial component of the Weed Risk Assessment system [25] used by Australia's Department of Agriculture, Fisheries and Forestry, which has been adapted for use in New Zealand [25], the Galapagos [26], and Hawaii and the Pacific Islands [27]. The accuracy of weed invasion risk assessments based on climate matching is crucial to the success of these programs at identifying and halting introductions of potentially noxious invasive species. Models for predicting the potential for plant invasions based on the climate of the native range have been developed for much of the woody introduced and native flora in North America [28], South Africa [10], Europe [10], [19] and Australia [29]. The same approach has been applied to numerous individual species of interest [e.g. 1], [3], [19], [30]–[32]. Hence, because climate matching is a component of so many weed risk assessment protocols, it is important to determine how valid the assumption of niche conservatism is among plant species.

Here, we use two broad classes of methods to test whether climatic niches differ between native and introduced populations of *Impatiens walleriana*, globally one of the most popular horticultural species. Native to tropical East Africa, *I. walleriana* has been found to establish and reproduce outside of human cultivation in locations as widespread as North and South America, the Pacific Islands, Australia, and New Zealand. To test hypotheses of niche expansion in this species, we use both approaches based on environments associated with raw occurrence data [as in 14], and model-based comparisons that take into account the sets of conditions available across different distributional areas, testing whether observed differences are greater than those expected based on the array of conditions available across the landscape.

## Methods

### Study species and occurrence data

We focused on the common horticultural annual *I. walleriana* (Balsaminaceae) as a test case for climate matching because it has been so widely translocated, and because temperate members of this genus have already received considerable attention as invasives [e.g. 33], [34], without comparable consideration of the far more numerous tropical species. The species has been planted globally for over a century [35]–[38], and ranked first among annual bedding plants in number of flats sold in the United States in the 1997 Census of Agriculture [39]. Most *I. walleriana* cultivars are seed-propagated [40].

We obtained occurrence data for *I. walleriana* from online herbarium databases using the search term “*Impatiens walleriana*” and the synonym “*Impatiens wallerana*.” Occurrence data from sites across the native range were also obtained from published floras [41]–[43]. For four collections from outside the United States missing latitude and longitude information, we estimated latitude and longitude to the nearest 0.01° based on reference to other studies at the same location [44], [45] For three collections within the United States, latitude and longitude were taken from the U.S. Census Bureau for the county of the collection [46]. Records indicating that a specimen came from a garden were excluded from analyses. Records were obtained from herbaria AAU, AD, BRI, CANB, CR, CUBA, DNA, FSU, INB, LPB, MEL, MO, NSW, NY, SI, and USF via the Global Biodiversity Information Facility. We based analyses on 27 native-range occurrences from Kenya, Malawi, Mozambique, Tanzania, and Zimbabwe, and 100 naturalized occurrence points from Argentina, Australia, Bolivia, Brazil, Colombia, Costa Rica, Ecuador, El Salvador, Honduras, Mexico, Nicaragua, Panama, Puerto Rico, United States of America (Florida), and Venezuela. The native range points cover the full known geographic range of the species, based on the best available botanical description [47]; however, whether they cover the full environmental range is a much more complex question that has not been examined in this contribution.

### Direct climate comparisons

To compare the climate space occupied by native populations directly with that occupied by introduced populations, we superimposed occurrence data on 7 “bioclimatic” variable grids (annual mean temperature, mean diurnal range, maximum temperature of warmest month, minimum temperature of coldest month, annual precipitation, and precipitation of wettest and driest months; 0.0833° resolution) [47]. We used the extract-to-point tool in ArcGIS 9.2 to obtain climate values for each occurrence record. Coastal records not coinciding with the terrestrial mask were assigned the values of the nearest cell on land. We tested for differences in the mean and breadth of native and introduced climate spaces using Welch's *t*-test and Levene's test for homogeneity of variance. All analyses were carried out in R version 2.9.2 [48].

### Ecological niche models

To complement the analyses of raw occurrence data, we developed ecological niche models for both the native and introduced ranges, using Maxent 2.2 and 3.2.1 [49], and used them to explore the question of niche shift or expansion in greater detail. We explored four approaches to creating ecological niche models to determine whether conclusions about niche expansion were consistent across methods, as follows. (1) We compared the predicted niche breadth of the native range when trained separately on native and introduced occurrence points using binary predictions of presence and absence (2–3). We compared niche breadth using standardized continuous suitability scores to control for effects of threshold suitability for predicted presence and differences in mean predicted suitability of habitat between models built using native and introduced points. We made these comparisons in both geographic and environmental space. Finally, (4) we used randomization tests to compare the observed differences in projected niche breadth between native and introduced ranges with the expected difference given the availability of environmental conditions in each area.

We buffered occurrence points by 2000 km, using these areas for training models and projecting results. Occurrence points that fell within the same 0.0833° grid cell were counted as single points, yielding a total of 22 native range points and 90 introduced range points. Dispersal in *Impatiens* species is via ballistic fruit, followed by secondary movement in water or caching by animals. Because of the possibility of long distance transport of floating in river systems, the 2000 km buffer is reasonable and sufficient to include relevant regions that have likely been accessible to the species over its period of residence on the landscape.

Comparisons of climatic niche breadth between distributional areas can be developed in either spatial [15] or environmental dimensions [12]. Models were built separately in the native and introduced buffered areas in Maxent with cumulative output using half the points within an area to test the model, and then projecting the model onto both native and introduced areas. We thresholded raw model output to binary using the least training presence threshold approach that emphasizes full prediction of the ecological niche of the species in question [50]. To compare the breadth of the modeled native and introduced niches, we compared predicted native ranges when trained separately on the native and introduced occurrence points. We extracted the values of the seven bioclimatic variables at each predicted presence grid cell in the predicted native range as a table, reduced the dimensionality of this dataset via principal components analysis to create orthogonal axes, and then compared the summed variances of axes as a measure of niche breadth between models [51]. We report on the first two axes, which explain approximately 70% of the variation.

A more comprehensive approach compares models while considering explicitly the availability of conditions in each distributional area [17], which are taken into consideration as follows. We do so via a set of randomization tests that compare the observed differences in niche breadth between models built using native and introduced occurrence points to the expected difference between models built using the same number of points randomly chosen from the broad geographic areas that represent the native and introduced range. We constructed models using the same seven bioclimatic data layers as well as 1-km resolution raster GIS data layers summarizing slope and aspect [52]. The Australian introduced range and the 39 Australian occurrence points were omitted from this analysis because slope and aspect data were not available for that region [52]. Models were built in Maxent using default settings with logistic output. Models were trained using only the study area for each set of occurrence points (native or introduced), and were then projected onto the available set of environments in both the native and introduced range (excluding Australia). Niche breadth was estimated by applying the inverse concentration metric of Levins [53] as implemented in ENMTools [54], [55] to the resulting sets of suitability scores, standardized so that minimum possible niche breadth within this space is 0 (indicating that only one grid cell in the geographic space has a nonzero suitability) and maximum niche breadth is 1 (where all grid cells are equally suitable). This metric does not require application of a threshold to produce predictions of presence and absence, but rather uses the continuous estimates of habitat suitability produced by Maxent directly.

We also compared the breadth of these models when projected into environmental space, as follows. Because comparisons in environmental space become exponentially more time-consuming as the number of variables increases, these comparisons were made with a reduced set of environmental variables. We chose the top three explanatory variables for each model based on Maxent contribution scores. Because slope was the variable with the highest Maxent contribution score for both native-range and introduced-range models, we ended up with a set of five variables. The minimum for each environmental variable was set to be the same as the minimum across the entire study area (introduced and native ranges combined), and maxima were selected similarly. Once the range was chosen, we divided each variable into 10 evenly spaced bins. An artificial grid was then constructed for each of the five variables so that every combination of the five variables was present exactly once, resulting in a grid of 10^5^  = 100,000 cells. New models were built in Maxent using the reduced and standardized set of variables, which were then projected onto the grid representing environmental space. These projected grids represented the estimated environmental suitability from native and introduced ENMs, regardless of whether or not those conditions were actually available in the area. Because all of the possible combinations of environmental variables are not represented in the geographic regions that occurrence points were drawn from, models would necessarily be required to extrapolate from that training data to predict suitability across the entire environmental space. We constrained this behavior by instructing Maxent to use “clamping”, a procedure which constrains predictions of environmental suitability so that environmental conditions that were not present in the training area do not produce extreme suitability estimates [49].

As discussed above, differences between native and introduced ranges in the suite of habitats available for species to occupy can lead to inferences of niche expansion when no evolutionary change has actually occurred. It is therefore important to compare the observed change in niche breadth to a null expectation based on the availability of habitat so we present the results of a randomization test intended to generate such a null expectation. For randomization tests, 100 pseudo-replicate data sets were constructed by choosing points randomly from the environmental background in the native and introduced areas, keeping sample sizes consistent with those from the actual data. Niche models were constructed for each pseudo-replicate, and niche breadth measurements for occurrences from the introduced and native ranges were compared, producing a distribution of expected differences in niche breadth given the available habitat in the two broad geographic areas. Because these areas were large and encompassed a great deal of environmental heterogeneity, pseudo-replicate niche models were expected generally to produce greater estimates of niche breadth than those from any actual species: it is unlikely that any biological species will be distributed completely without regard to environmental variables. Nevertheless, the distribution of expected overlaps generated by this randomization test is informative, as it estimates expected differences in niche breadth if species' environmental tolerances were uniform across all combinations of environmental variables, and dispersal to all regions considered “background” were possible.

## Results

### Direct climate comparisons

**Figure 1:**
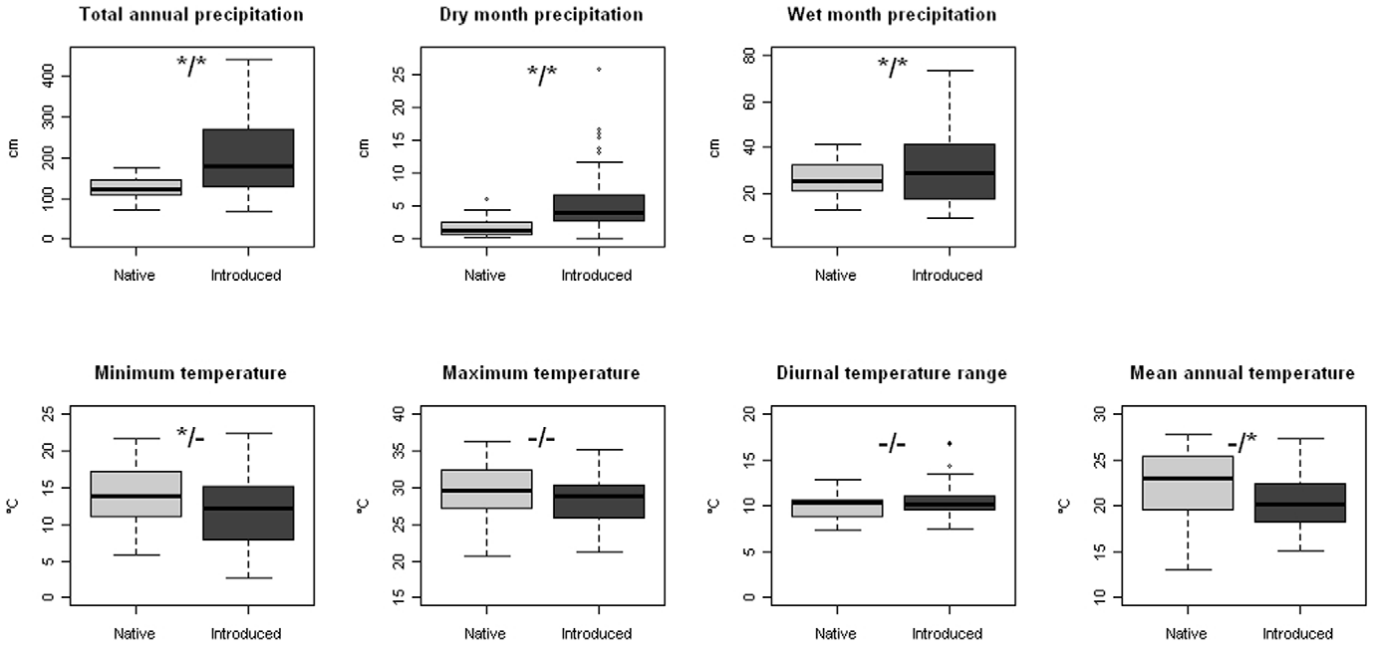
Example climatic parameters of *Impatiens walleriana* across localities sampled from the native and introduced ranges. * indicates a significant difference (mean/breadth); - indicates nonsignificant differences. Shown are the median (line) ±interquartile range (boxes) ±1.5 times the interquartile range (whiskers).

Introduced populations of *I. walleriana* showed a broader climatic range, being found in areas far wetter than the native range (Fig. 1). Introduced populations occurred in areas with higher annual precipitation (t = 7.19, df = 124.01, p<0.001), and higher precipitation in the driest (t = 5.67, df = 104.22, p<0.001) and wettest (t = 2.01, df = 87.68, p = 0.047) months. The breadth of the climatic range also increased in introduced populations for these three factors (annual precipitation: F = 20.727, df = 1, 25, p<0.001; dry month precipitation: F = 4.3726, df = 1, 25, p = 0.039; wet month precipitation: F = 12.932, df = 1, 25, p<0.001). The increased climatic range comes almost exclusively from the presence of introduced populations in wetter areas, with few introduced populations occurring in locations drier than the native range.

Temperature differences between introduced and native populations were more subtle, but overall suggest that introduced populations occur in cooler climates than native populations. The minimum temperature was significantly lower in the introduced range (t = −2.46, df = 46.11, p = 0.018). The range of mean annual temperatures within the introduced range was significantly narrower than that of the native range (F = 4.06, df = 1, 25, p = 0.046), and the mean temperature was lower, but not significantly so (t = −1.83, df = 34.67, p = 0.075). We found no significant differences in the mean or breadth of maximum temperature (mean: t = −1.53, df = 36.64, p = 0.135; F = 1.82, df = 1, 25, p = 0.179) or diurnal temperature range (mean: t = 1.45, df = 44.29, p = 0.15; breadth: 0.049, df = 1, 25, p = 0.825).

**Figure 2:**
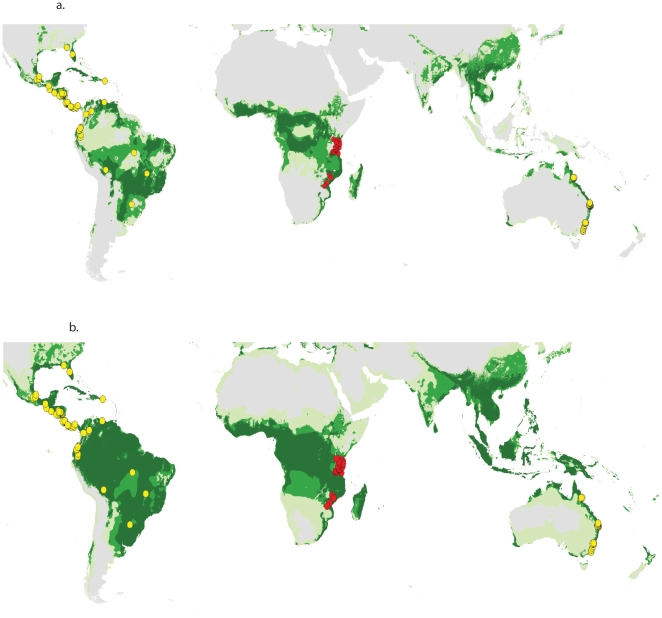
Climate matching for *I. walleriana* based on the climate of (A) native populations and (B) both native and introduced populations. Green shades indicate land surfaces with five, six and seven climate variables that fall within the native climate space, with darker shades indicating more matching climate variables. Gray indicates land surfaces with four or fewer climate variables in common with the range used to develop the model. Native localities used are shown in red, and the introduced localities in yellow.

Using the ranges of these seven climatic factors observed for the species in the native range to identify similar locations for *I. walleriana* globally, 6.1% of the global terrestrial land surface matched all seven variables, and 13.2% matched six or more variables (Figure 2a). Forty-three out of 100 introduced occurrence localities matched fewer than six of the seven native climate variables, indicating that simple climate matching failed to predict naturalization success. Fourteen of 100 occurrence localities matched fewer than five native variables, primarily localities in Australia and Costa Rica that were wetter than the native range. Expanding parameter estimates to include both introduced and native populations identified 18.3% of the land surface matching all seven variables and 24.9% matching at least six variables (Fig. 2b).

### Ecological niche models

**Figure 3:**
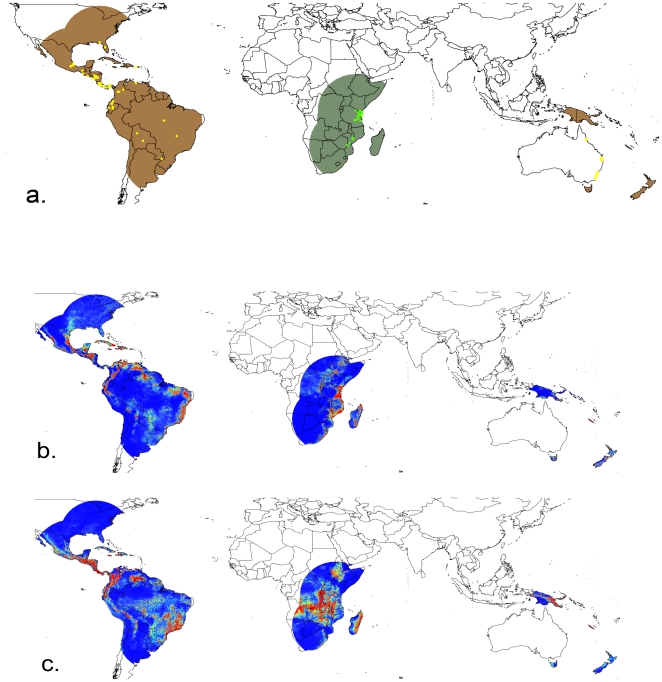
Study area and Maxent model predictions. Panel 3a shows the study region for native (green) and introduced (brown) study areas. Occurrence points are shown for native (bright green) and introduced (yellow) localities. Panels 3b and 3c show Maxent models built for native and introduced specimens, respectively. Warmer colors indicate habitat that is more suitable according to the model.

We developed ENMs for both the native and introduced ranges (Figure 3a–c). When models of each range area were used to predict the other range, predicted patterns of potential occurrence were similar. Considering the semi-continuous nature of the climate variables (i.e., as integers), we can measure niche breadth provisionally as numbers of unique combinations of environmental variables: the introduced range models predicted grid cells in the native region with 44,862 bioclimatic combinations, while native range models predicted 50,591 bioclimatic combinations in the native range of the values for the 7 layers we used. PCAs summarized variation in the seven bioclimatic variables, with the first two axes explaining 70.9% of the variance in the introduced-trained native range, and 63.7% of the native-trained native range. The sum of the variances calculated independently along each axis of the environmental space was greater when the model was trained on native-range occurrences (5.237) than on introduced-range occurrences (4.753), the opposite of the direction expected if the introduced populations had greater breadth.

In the standardized-space comparisons, niche breadth estimated based on occurrences from the introduced range was considerably greater than that based on native occurrences, both in geographic space (standardized inverse concentration of 0.175 using occurrences from the introduced range; 0.043 using records from the native range) and environmental space (inverse concentration of 0.576 using introduced range; 0.139 using native range). However, randomization tests, which estimated the effects of differential habitat availability on niche breadth for native and introduced populations, were unable to reject the hypothesis that the observed niche expansion resulted solely from differences in available habitat between native and introduced ranges. In fact, the observed niche expansion in the introduced range was significantly less than that expected given the distribution of environmental variables available, as seen by comparing the observed difference in niche breadth between models built using native and introduced occurrence points to the distribution of expected overlaps from models built using randomly drawn points from those geographic areas (*p*<0.01).

## Discussion

### Interpretation of results

Analysis of raw climatic variables associated with occurrence points suggested that naturalized populations of *I. walleriana* occupy climates wetter than are found in the native range, while more controlled comparisons based on ecological niche models did not support the idea of a niche expansion in introduced populations. Although it is clear that introduced populations occur in locations that are wetter and cooler than the native populations, this difference appears to result solely from greater availability of such habitats in the introduced range. At the very least, this explanation cannot be rejected in favor of the idea of a real shift in habitat preference.

African species of *Impatiens* tend to favor moist areas [56]. *I. walleriana* follows this trend, as is indicated by the high frequency with which herbarium records mention the presence of a creek, stream, or ditch. Within its native range, *I. walleriana* occurs in the wetter parts of the region. Outside of its native range, *I. walleriana* has expanded into areas that are more humid and with higher annual precipitation than any part of East Africa. These observations suggest that *I. walleriana* can tolerate such conditions, but that apparent climatic niche differences may be deceptive. Additionally, the importance of environmental conditions (e.g. moisture) that can vary greatly across short distances to the distribution of *I. walleriana* suggests that the resolution of existing global environmental datasets may be too coarse to accurately describe the species' ecological niche.

The term “cryptic climatic adaptation” was coined by Panetta and Mitchell [9] to describe the ability some species may possess to grow in climates outside that of their native range. Cryptic climate adaptation may be an underappreciated factor in examinations of climatic niche conservatism [57], [58], because some individuals within populations may be preadapted to more extreme climates [59]. The mechanism behind the observed expansion of *I. walleriana* into wetter climates is likely not adaptation to novel conditions – but rather residual adaptation from formerly predominant conditions that still occur within its current native range. *I. walleriana* in its current native range may be growing under a more restricted set of conditions – a sort of climatic cage – from which anthropogenic transport as an ornamental provided an escape. Since the Last Glacial Maximum, East Africa has been alternatively wetter and drier than at present [55], but present conditions are skewed toward the dry end of the spectrum.

These observations do not mean that climate does not limit the establishment and spread of *I. walleriana* in either its native or its introduced ranges. For example, despite constant introduction pressure throughout the continental United States, populations have only established outside of cultivation in Florida, suggesting that cold winters limit establishment in temperate climates [60]. This difference is more or less equivalent to the distinction between the fundamental ecological niche and the existing fundamental ecological niche, in that the same niche may have very different “existing” manifestations on different landscapes, in the present case between the native and introduced distributional areas of the species in question.

### Limitations of niche models for predicting invasion in novel climates

This suite of analyses illustrates the importance of careful consideration of geographic areas of analysis and extents of applicability in niche modeling exercises. Soberón and Peterson [4] offered a heuristic framework for understanding distributions of species: species occur in areas that (1) are within the set of abiotic conditions that are appropriate to maintain populations, (2) present the appropriate set of biotic conditions, and (3) are within the species' dispersal and colonization reach over relevant time periods.

This latter consideration – the area that species is able to “explore,” effectively sampling conditions via dispersal and either establishing populations or not – proves central to developing niche models and to consideration of niche shifts such as that presented here. Outside of this area of exploration, the species is absent, but may be absent in spite of fully suitable conditions (hence the potential for invasiveness when species cross dispersal barriers). In this case, models fitted over extents including areas outside of this region will be confused by these uninhabited suitable areas. More directly relevant to the present case, application of climate-based niche models to predict potential ranges is only suitable at sites presenting climatic conditions that have been previously explored by the species. Model predictions outside of these conditions are extrapolations, and will not be reliable or interpretable.

The full fundamental climate niche of *I. walleriana* cannot be determined from its native range alone because its distribution there is modified by biotic and dispersal factors, and particularly by the limited set of environments represented there. When attempting to determine whether an introduced species is at risk of establishing in a specific location, it is thus important to include existing knowledge about a plant's introduced range. Not surprisingly, studies of invasive species have found that that models parameterized from the introduced range [18] or combined native and introduced range [13] yield more accurate predictions than models parameterized from the native range alone. However, introduced populations may be out of distributional equilibrium – i.e. not inhabiting the entirety of the habitable area in the region. In such cases, introduced-range models may underestimate the species' ecological potential. Whether differences in climate niches between native and introduced ranges result from differences in climate availability or evolution of the climate niche, data drawn from a wider climate range would be expected to yield more informative results about the breadth of the climate niche.

For biosecurity, it has been hoped that modeling native range distributions will provide a sort of lower bound on the environmental suitability of new habitat [e.g. 25]–[27]. At best, these approaches have usefulness in only one direction – a high value of suitability may indicate a high potential for invasiveness, but low values may indicate simply a lack of information. Critically, lack of match between the native range and the range of proposed introduction ignores the unknown nature of species' responses to climatic conditions not represented in the native range. That is, if the “other” range area includes environmental conditions not represented on the native range, projections of models to those landscapes consist of genuine “extrapolation,” and will be prone to diverse problems of how model rules extend into unknown environmental territory. In this sense, although using native range data for training such models is better than doing nothing, it is valid only to the degree that environments there are representative.

### Reflections on niche change during invasions

Several recent studies have attempted to assess whether niches differ between native-range and introduced-range populations of species [14], [15], [23]. In these studies, the emphasis has been on evolutionary changes in niche dimensions or in ecological changes in the realized niche owing to changes in the biotic environment [61]. Most such studies have relied on comparisons of environmental conditions associated with raw occurrence data, with the argument that such data represent more accurately the true patterns underlying the species' distribution. In each case, the conclusion has been that niche shifts are indeed occurring [23].

This study, to be frank, began along the same lines, comparing environmental conditions associated with occurrences of a species on its native and introduced ranges, concluding that the niche of *I. walleriana* had expanded in tandem with the invasion process. However, upon quantitative consideration of the environmental contexts within which these populations are distributed, the conclusion changed: introduced populations of *I. walleriana* indeed occupy a broader set of environments than native-range populations, but this difference likely reflects the limited spectrum of environments manifested on the species' native range in East Africa, and not genuine niche expansion.

The reasons for the discrepancy between the results of the two sets of approaches to testing niche conservatism are at least two-fold. In the first place, sample sizes may be inflated considerably and artifically in raw occurrence comparisons owing to spatial autocorrelation and consequent non-independence of points. Second, the randomization tests presented here permit us to control for the area accessible to the species via dispersal, equivalent to the mobility constraint of Soberón & Peterson [4]. If not taken into account, environmental differences between the areas accessible to the species on its two ranges may appear to result from niche shifts when they have not, in reality, changed. To determine if niche shifts are occurring in biological invasions, it is essential to assess environmental differences between native and introduced ranges.
